# Bifocal Radial Fracture/Dislocation and Distal Ulnar Fracture—A Rare Case of Proximal Forearm Instability Not Yet Classified and Literature Review

**DOI:** 10.3390/jcm14134694

**Published:** 2025-07-02

**Authors:** Michele Dario Gurzì, Giacomo Capece, Guido Bocchino, Alessandro El Motassime, Rocco Maria Comodo, Massimiliano Nannerini, Giulio Maccauro, Raffaele Vitiello

**Affiliations:** 1 Aurelia Hospital—U.O.C. Orthopaedics and Traumatology, 00165 Rome, Italy; mgurzi@gmail.com (M.D.G.); massinannerini@gmail.com (M.N.); 2 Department of Orthopedics, Ageing and Rheumatological Sciences, Fondazione Policlinico Universitario A. Gemelli IRCCS, Largo Agostino Gemelli 8, 00168 Rome, Italy; guido.bocchino@hotmail.it (G.B.); alessandroelmotassime@gmail.com (A.E.M.); roccocomodo96@gmail.com (R.M.C.); giulio.maccauro@unicatt.it (G.M.); lele.vitiello@gmail.com (R.V.); 3 Pellegrini Hospital—U.O.C. Orthopaedics and Traumatology, 80134 Naples, Italy; 4 Department of Orthopedics and Geriatric Sciences, Catholic University of the Sacred Heart, Largo Francesco Vito, 8, 00168 Rome, Italy

**Keywords:** forearm fracture, Monteggia, Bado, Essex–Lopresti, trauma, orthopedics

## Abstract

Introduction: Monteggia fractures, first described by Giovanni Battista Monteggia, involve a fracture of the proximal ulna with anterior dislocation of the radial head. Bado’s 1967 classification divides these injuries into four types. Rare mixed patterns exist, overlapping with other forearm injuries such as Galeazzi and Essex–Lopresti lesions. These complex fractures/dislocations pose significant diagnostic and therapeutic challenges and are not adequately represented in current classification systems. Methods and Case Presentation: We report the case of a 56-year-old woman with a complex forearm injury sustained from a fall, presenting with radial head fracture/dislocation, mid-shaft radial fracture, distal ulna fracture, and ulnar collateral ligament rupture. Intraoperative imaging confirmed DRUJ stability and partial interosseous membrane disruption. Surgical management included radial head prosthesis implantation, radial shaft fixation with an anatomical locking plate, intramedullary nailing of the distal ulna, and ligament reconstruction. At two-year follow-up, the patient demonstrated full recovery of elbow flexion–extension and satisfactory forearm function. A narrative literature review was also conducted, focusing on hybrid injury variants. Results: Intraoperative examination under anesthesia revealed good elbow stability with 130° flexion, 15° extension lag, and forearm pronation/supination of 70°/60°. An initial Mayo Elbow Performance Score (MEPS) of 65 was recorded, limited by range of motion and stability. Pain during passive mobilization was mild, with a Visual Analogue Scale (VAS) score of 3/10. Postoperative recovery included 15 days of immobilization followed by structured rehabilitation. At two years, the patient regained full elbow flexion–extension but had residual deficits in pronation–supination, attributed to pre-existing conditions. Conclusions: This case illustrates a previously unreported hybrid Monteggia variant, combining features of Monteggia, Galeazzi, and Essex–Lopresti injuries. It highlights the limitations of current classification systems and supports the need for an expanded diagnostic framework. Successful management required a multidisciplinary surgical approach tailored to the injury’s complexity. Further studies are warranted to refine classification and treatment strategies for these rare combined injuries.

## 1. Introduction

Fractures/dislocations of the forearm involving both the radius and ulna, particularly when associated with joint instability, constitute an uncommon yet highly challenging spectrum of orthopedic injuries [1]. These lesions frequently demand complex diagnostic assessments and a nuanced, often individualized, surgical strategy to achieve anatomical reconstruction and restore function. Among these injuries, Monteggia fracture/dislocation represents a classical subtype, first described by Giovanni Battista Monteggia in 1814, and later classified into four types by José Luis Bado in 1967, based primarily on the direction of radial head dislocation [1,2]. Type IV Monteggia lesions, which involve fractures of both the proximal radius and ulna with anterior dislocation of the radial head, are the rarest in adult populations, accounting for less than 3% of all Monteggia variants [3,4]. While the Bado classification [1] provides a useful framework for identifying and guiding the treatment of these injuries, real-world clinical scenarios frequently present with more complex patterns. Atypical fractures/dislocations that exhibit features from multiple forearm injury types challenge existing taxonomies and highlight the limitations of rigid classifications. In particular, some cases combine features of other distinct injury types, such as Galeazzi and Essex–Lopresti lesions, complicating classification and treatment.

In the last decades, several authors have attempted to refine and expand the classification of atypical Monteggia variants by proposing subtypes based on clinical, radiological, and biomechanical features [5]. Additionally, decision tree models have been explored to aid the diagnostic process and therapeutic decision-making in complex forearm traumas, offering a more flexible and adaptive approach compared to traditional rigid classifications [6]. These clinical tools facilitate the identification of associated lesions, such as ligamentous injuries and interosseous membrane involvement, and support the planning of targeted surgical interventions. However, these models are not yet widely adopted nor consistently validated, and the literature remains fragmented regarding shared classification systems for hybrid forearm injuries. This gap underscores the urgent need for more comprehensive and dynamic classification frameworks capable of encompassing complex cases and emerging variants.

The Galeazzi fracture is defined by a fracture of the distal radial diaphysis with associated dislocation of the distal radioulnar joint (DRUJ) [7], while the Essex–Lopresti lesion comprises a comminuted radial head fracture, disruption of the interosseous membrane (IOM), and DRUJ dislocation [8]. Both injuries are associated with longitudinal instability of the forearm, and the diagnostic oversight of associated soft tissue injuries can lead to poor functional outcomes, including chronic instability, pain, and reduced range of motion [9,10].When combined or hybrid patterns occur, exhibiting elements from Monteggia, Galeazzi, and Essex–Lopresti lesions, therapeutic management becomes even more complex. These cases demand a multidisciplinary approach, often involving radial head arthroplasty, ligamentous reconstruction, and stable fixation of both forearm bones [11].

The rarity of these hybrid injuries has led to a limited body of literature and a lack of standardized treatment protocols. Isolated case reports and small case series remain the primary sources of information. However, these can still provide meaningful clinical insights when interpreted through structured frameworks. Integrating literature via narrative reviews allows for the synthesis of diverse findings and the identification of shared therapeutic principles, despite heterogeneity in case presentations and surgical techniques.

The present study pursues a dual objective. First, we report an unusual clinical case involving a hybrid fracture/dislocation pattern that exhibits overlapping features of Monteggia Type IV, Galeazzi, and Essex–Lopresti lesions. The patient was treated successfully through a multidisciplinary surgical strategy that addressed both osseous and ligamentous components of the injury. Second, we conduct a narrative review of the current literature on complex forearm fractures/dislocations, with particular attention to surgical principles, implant selection, and strategies for restoring longitudinal and rotational stability.

Given the increasing recognition of hybrid patterns that defy traditional taxonomies, there is a compelling need to expand or adapt existing classification systems. In this regard, we propose a preliminary conceptual framework for categorizing such injuries, tentatively termed “Hybrid Monteggia Variants”. These could be subtyped based on the concomitant involvement of key elements from other well-described patterns, such as distal radioulnar joint disruption, interosseous membrane injury, or radial head comminution, alongside proximal ulna fractures and radial head dislocation. While not a formal classification, this conceptual schema aims to encourage structured recognition and facilitate shared terminology in future clinical reporting and research.

In summary, this article provides a detailed case report and a literature-based synthesis on the management of complex forearm fractures/dislocations. By highlighting the diagnostic and therapeutic challenges of a rare Monteggia-like injury with overlapping characteristics, we aim to contribute to the evolving understanding and classification of hybrid forearm injuries, while offering practical insights into their surgical management.

## 2. Methods and Case Presentation

### 2.1. Case Presentation

A 56-year-old woman presented to the emergency department with acute pain and visible deformity of her left upper limb following a fall several hours earlier. The incident occurred while she was attempting to lift a heavy metal shutter, and in the process, she braced herself with her left arm to cushion the impact. The patient’s medical history was notable for osteoporosis and hypertension, as well as a longstanding limitation in forearm pronation and supination resulting from a traumatic injury sustained during adolescence.

**Figure 1:**
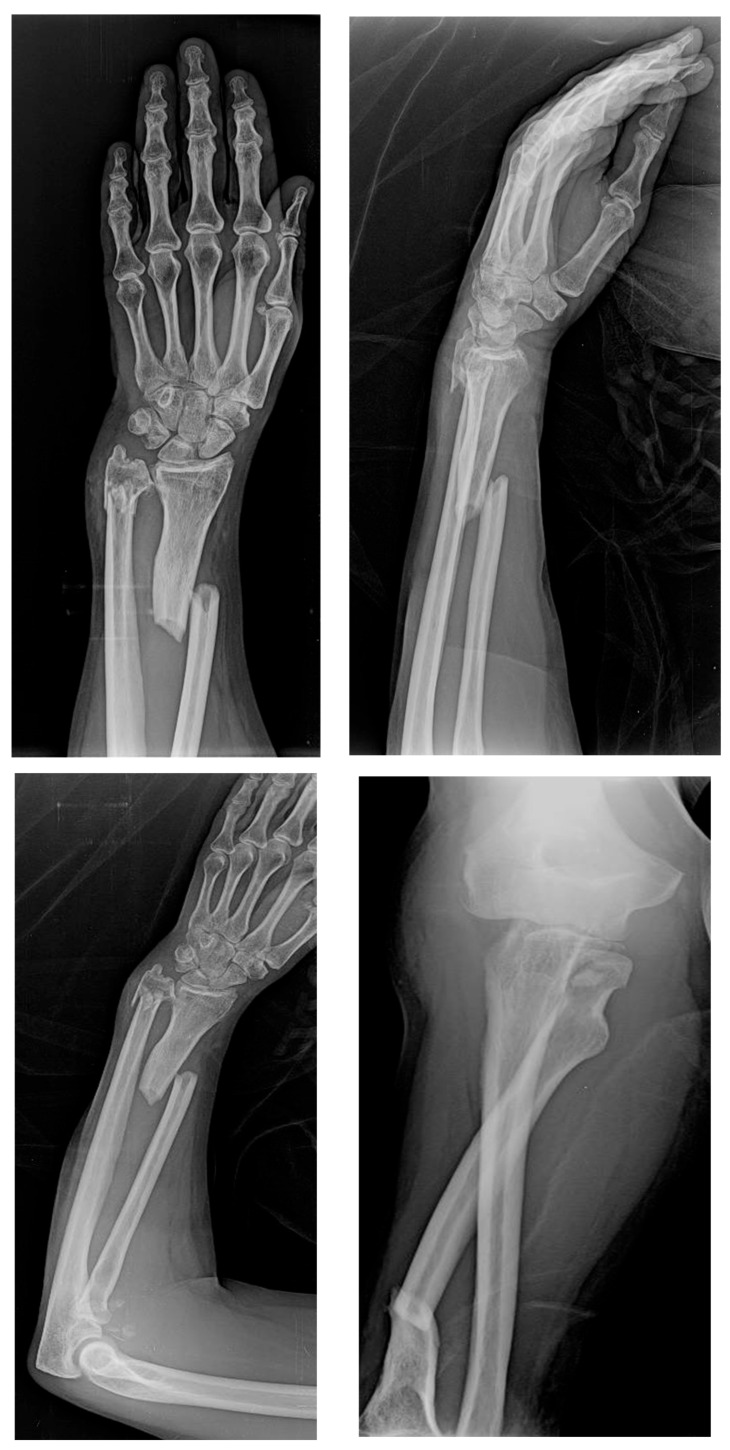
X-rays performed at the emergency room entrance.

**Figure 2:**
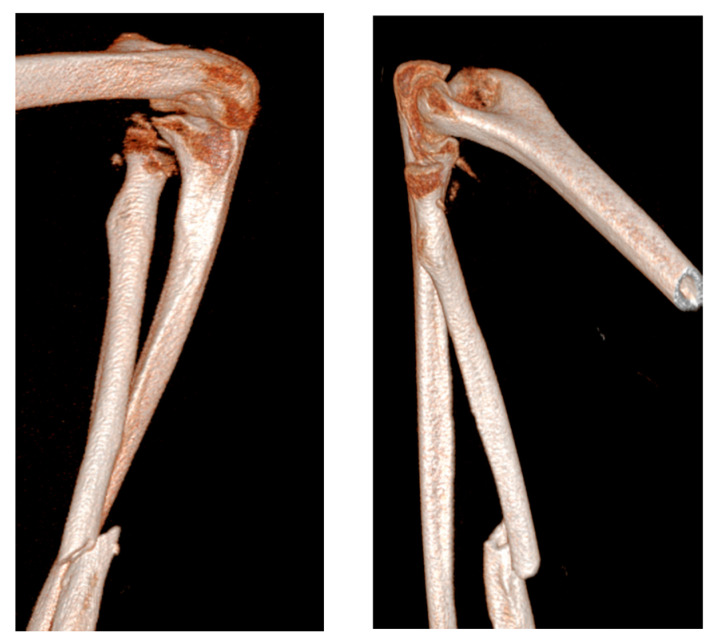
CT-based 3D model performed at the emergency room entrance.

Upon physical examination, the patient exhibited a gross deformity of the left arm, with tenderness localized to the olecranon and wrist regions. The limb demonstrated complete functional impairment, with limited movement and significant swelling. Radiographic evaluation, including urgent X-rays, revealed a comminuted fracture of the distal third of the radius with dorsal displacement of the distal fragment and a comminuted fracture of the distal ulna metaphysis. Additionally, there was a multi-fragmentary radial head fracture, accompanied by partial detachment of the articular head, which raised concern for significant instability (Figure 1 and Figure 2). Based on these findings, a diagnosis of complex forearm fracture/dislocation with suspected longitudinal instability was made.

**Figure 3:**
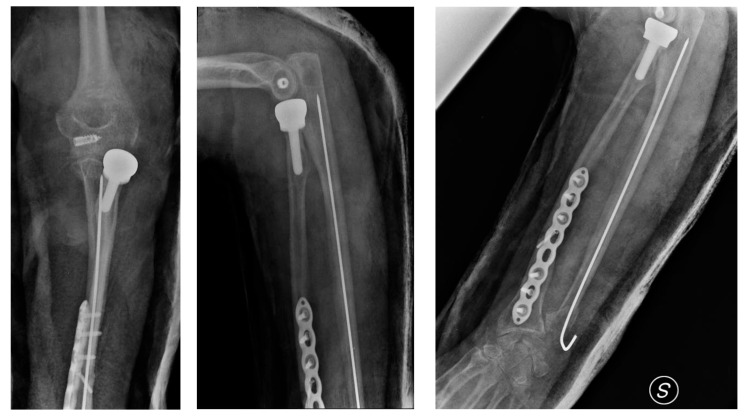
Postoperative follow-up X-rays.

The following day, the patient underwent surgical intervention. The procedure included open reduction and internal fixation (ORIF) of the radial fracture using an anatomical locking plate, as well as closed reduction and intramedullary fixation of the ulnar fracture with a Steinmann pin. Given the extent of the radial head fracture, a prosthetic radial head replacement was performed. Ulnar collateral elbow ligament reconstruction was also carried out to restore elbow stability. Intraoperative assessment confirmed the integrity of the distal radioulnar joint (DRUJ), although there was partial damage to the interosseous membrane, which was carefully addressed during surgery (Figure 3).

Under anesthesia, passive ROM reached 130° flexion, with a 15° extension deficit and pronation/supination of 70°/60°. Intraoperative joint stability was satisfactory in both varus and valgus stress tests, as well as during axial loading.

To provide an early functional baseline, a preliminary Mayo Elbow Performance Score (MEPS) of 65 was estimated, mainly limited by motion and stability parameters. Pain was assessed using a Visual Analogue Scale (VAS), scoring 3/10 under passive mobilization. These metrics, though initial, offer a valuable reference for postoperative rehabilitation planning and future clinical assessments.

**Figure 4:**
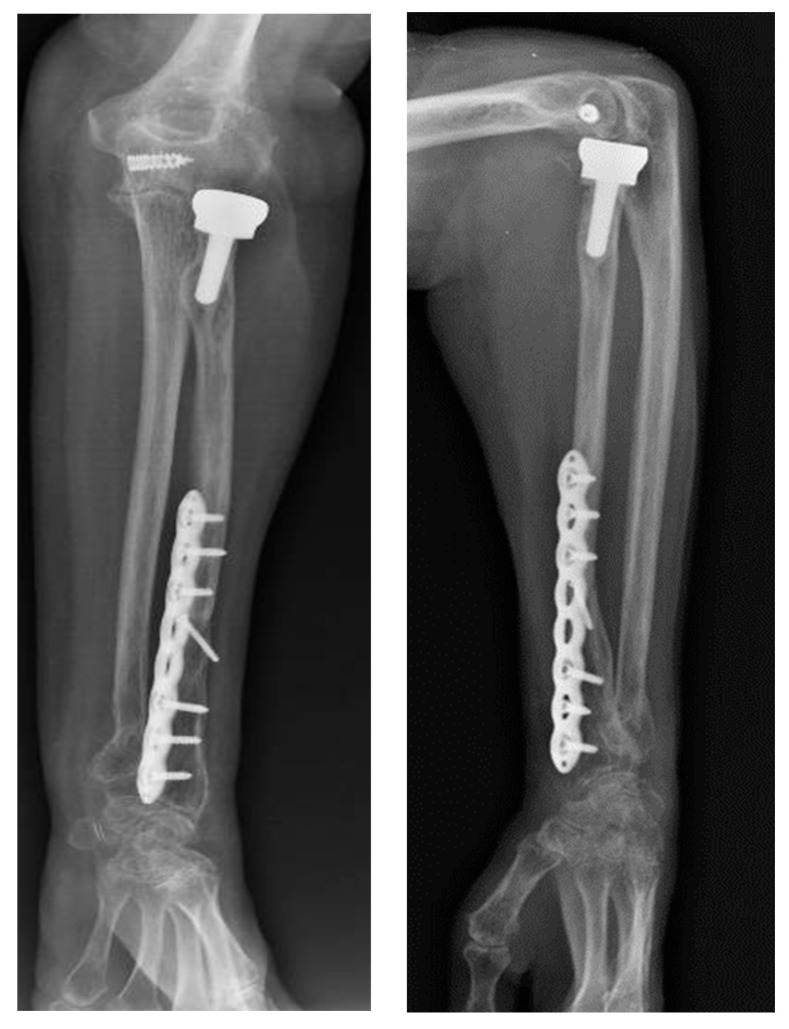
Two-year follow-up X-rays.

Postoperatively, the arm was immobilized in a neutral-flexed position using a BAM-type plaster splint for 15 days. Following immobilization, the patient began a regimen of protected active and passive mobilization to restore range of motion. A targeted physiotherapy protocol was initiated one month after surgery to support the recovery of elbow and forearm function. At the two-year follow-up, the patient had achieved full recovery of elbow flexion and extension. However, some limitations in forearm pronation and supination persisted, likely due to her pre-existing condition, which was further investigated with a repeated X-ray (Figure 4). The timeline of findings, procedures, and outcomes is summarized in Table 1.

### 2.2. Narrative Review Strategy

A structured narrative review of the literature was conducted to investigate complex forearm fractures/dislocations, with a particular focus on hybrid variants. These types of injuries, though distinct in their characteristics, can overlap in their clinical presentations, posing significant diagnostic and therapeutic challenges. A central goal of this review was to synthesize current knowledge surrounding the mechanisms of injury, treatment strategies, outcomes, and complications, aiming to highlight gaps in the literature and suggest future areas for research.

To gather relevant sources, an extensive literature search was performed across multiple databases, including PubMed/MEDLINE, Embase, and Scopus. The inclusion criteria were based on articles published up to 2024 in English, Italian, French, or Spanish, as these languages are commonly used in orthopedic literature and encompass a wide range of clinical research. Only studies that addressed complex forearm fractures, specifically involving the radius and ulna, were included. These studies encompassed clinical case reports, retrospective cohort studies, narrative reviews, surgical technique papers, and brief communications, all of which were evaluated for their relevance to the topic of mixed or complex forearm injuries.

Inclusion criteria for studies involved explicit mentions of therapeutic management approaches, surgical techniques, long-term prognosis, and complications associated with these fractures. Specifically, the focus was on articles discussing hybrid fractures/dislocations, cases that involved both radial head fractures and ulnar injuries, and injuries that presented as rare variants of the more common Monteggia, Galeazzi, or Essex–Lopresti fractures. Articles with a focus on less common aspects, such as interosseous membrane involvement and ligamentous disruptions, were also prioritized, given their potential role in the pathophysiology of complex forearm injuries.

The literature search was guided by a defined set of keywords that were carefully selected to capture the broad spectrum of topics relevant to the review. These included terms such as “Monteggia fracture,” “Monteggia variant,” “Galeazzi fracture,” “Essex–Lopresti lesion,” “complex forearm fracture-dislocation,” “radial head prosthesis,” “ligament reconstruction,” “ulna fixation,” and “interosseous membrane injury.” These terms were used in various combinations across the databases to ensure a wide coverage of the subject matter.

The strategy was designed to prioritize high-quality clinical studies and avoid being overly influenced by small case series or studies with methodological limitations. The review was conducted without a quantitative meta-analysis, as the primary aim was not to pool data for statistical analysis but rather to qualitatively synthesize the findings of various studies. This qualitative approach was preferred due to the heterogeneity in study designs, populations, and outcome measures present in the available literature. By focusing on synthesizing the evidence, the narrative review aimed to provide a more clinically relevant and contextually rich overview of the management strategies and outcomes associated with complex forearm fractures/dislocations.

This review aimed to consolidate knowledge from clinical case reports and studies, integrating evidence on the efficacy of surgical techniques (such as radial head prosthesis implantation and ligament reconstruction) and discussing the challenges associated with different approaches. Special emphasis was placed on treatment modalities that could address the unique combination of fractures found in hybrid injuries, such as radial and ulnar fractures occurring together with ligamentous and membrane injuries.

By employing a narrative review methodology, this paper aimed to draw meaningful conclusions about current practices, while acknowledging the limitations posed by the lack of randomized controlled trials or large-scale prospective studies in the management of these complex injuries. It is hoped that this review will provide a valuable resource for clinicians dealing with similar cases and will stimulate further investigation into refining treatment strategies and improving patient outcomes for this challenging injury type.

To ensure methodological rigor, we applied structured selection criteria and transparently documented the search process. Two independent reviewers screened titles and abstracts for relevance, followed by full-text assessment. Any discrepancies were resolved by consensus. Although formal risk-of-bias tools were not applied, studies were assessed based on the clarity of case description, completeness of treatment data, and adequacy of follow-up. A summary table (Author, Year, Injury, Treatment, Outcomes) has also been included to synthesize the main characteristics and findings of the reviewed studies (Table 2).

## 3. Discussion

The case presented in this report highlights a rare and complex subtype within the already uncommon group of fracture/dislocation syndromes [8]. Monteggia fractures/dislocations represent only 0.7% of all elbow fractures/dislocations. In this clinical scenario, we encountered an extremely rare injury, previously unreported in the medical literature, involving a combination of fracture/dislocation and elbow instability.

At the time of admission, the patient reported having self-reduced the elbow dislocation. The exceptional nature of this case lies in the coexistence of fracture/dislocation and marked joint instability, evidenced by rupture of the ulnar collateral ligament and a distal ulna fracture. The only remaining structural support was a partially preserved interosseous membrane. During surgery, it became clear that stability could not be restored by simple fixation of the radial shaft fracture. Instead, elbow and forearm stability was achieved only after radial head prosthesis implantation, reconstruction of the ulnar collateral ligament, and fixation of the distal ulna fracture.

This case illustrates the importance of a thorough diagnostic approach in the presence of complex forearm fractures, with attention to both osseous and ligamentous injuries. It also emphasizes the need for prompt, targeted surgical intervention and early rehabilitation to restore range of motion and prevent stiffness in the elbow and wrist [10].

Early rehabilitation plays a critical role in optimizing functional outcomes following complex forearm fractures/dislocations. In our case, passive ROM exercises for the elbow and wrist were initiated under supervision within the first postoperative week, after removal of the surgical drain and stabilization of soft tissues. The initial focus was on pain-free flexion–extension of the elbow, gentle forearm pronation–supination, and active-assisted wrist movements, avoiding valgus stress to protect the repaired ulnar collateral ligament. By the third postoperative week, the patient progressed to active ROM exercises, including gradual loading with a soft ball and pronation–supination in midrange positions. At six weeks, isotonic strengthening exercises were introduced, particularly targeting grip strength, brachioradialis, and pronator teres, along with neuromuscular retraining to restore coordinated forearm and elbow function. A full return to daily activities was achieved at 12 weeks, with sports-specific training introduced after 16 weeks, depending on pain and functional stability.

Monteggia fractures, although rare, represent complex injuries of the proximal forearm often associated with radial head dislocation. Among the four patterns classified by Bado, type IV, characterized by fractures of both the proximal radius and ulna with radial head dislocation, is the least common, accounting for approximately 3% of adult Monteggia injuries [3,4].

The management of Bado type IV injuries is particularly challenging, especially when features mimic Galeazzi or Essex–Lopresti lesions, including disruption of the distal radioulnar joint and interosseous membrane. In such cases, the surgical strategy must focus on restoring the longitudinal stability of the radius, achieving anatomical alignment of the ulna, and addressing the radial head injury through fixation or prosthetic replacement.

Weber et al. [11] reported that patients with complex Monteggia-like lesions often require multiple surgical interventions due to complications such as nonunion of the ulna and limited elbow mobility. Rigid fixation of the ulna, ideally with locking compression plates, is critical to ensure stability and promote proper bone healing.

Tille et al. [12], in a retrospective review of 35 cases, found that fracture location within the ulna significantly influenced outcomes. Fractures closer to the elbow joint, particularly those involving the coronoid process, were associated with poorer functional results, while the involvement of the radial head was less prognostically significant.

Several authors, including Klug and Jungbluth [13,14], have underlined the central role of radial head management in complex forearm injuries. Klug et al. demonstrated that the choice between osteosynthesis, prosthetic replacement, or resection has a significant impact on clinical outcomes, particularly in comminuted Mason type III fractures. Similarly, Ring et al. [16] emphasized the value of radial head prosthesis when primary fixation is not feasible.

Residual instability following bony fixation often necessitates repair or reconstruction of the lateral collateral ligament complex, especially in Bado type IV injuries, where elbow biomechanics are severely compromised [17,18]. Jungbluth et al. [14] also pointed out that overlooked soft tissue injuries commonly cause suboptimal outcomes. In particular, injuries to the interosseous membrane may contribute to longitudinal instability, as highlighted by Biewener et al. [19].

Surgical sequencing plays a key role in the management of these injuries. O’Driscoll and Jupiter [15,20] recommend a systematic approach: stabilize the ulna first, assess radial head alignment, and then address ligamentous injuries, with intraoperative fluoroscopy used to confirm reduction and stability.

Delayed union or nonunion of the ulna and persistent instability are among the most common complications. Although no high-level evidence supports one specific treatment algorithm, locking compression plates appear to offer superior mechanical stability compared to other fixation methods [21]. Complications related to radial head osteosynthesis (13%) and persistent joint instability (12%) are also frequent causes of revision surgery in Monteggia injuries. Posterolateral instability of the radial head remains a key contributor to poor outcomes, especially in the setting of comminuted Mason III fractures. Klug et al. [13] found that reconstruction yielded better results than resection or arthroplasty, though further research is necessary to determine whether prosthetic replacement or resection leads to more favorable long-term outcomes.

In conclusion, Bado type IV Monteggia fractures require a multidisciplinary and individualized surgical approach. Ulna fixation with locking plates, careful management of the radial head, and repair of ligamentous structures are essential to optimize functional recovery. Although these lesions are rare and evidence is limited, clinical experiences from authors such as Weber, Klug, and Korner [11,13,17] provide valuable guidance for management.

The rarity and complexity of the present case underscore the limitations of existing classification systems, such as Bado’s, which do not account for hybrid patterns involving features of Monteggia, Galeazzi, and Essex–Lopresti lesions. These overlapping presentations challenge rigid diagnostic frameworks and call for a more flexible, integrative approach. In light of this, we propose a preliminary conceptual framework for recognizing these atypical injuries under the umbrella of “Hybrid Monteggia Variants.” This term may encompass cases where a Monteggia-type pattern coexists with elements such as distal radioulnar joint disruption, interosseous membrane injury, or comminuted radial head fractures.

While not intended as a formal classification, this schema encourages more precise characterization of complex cases and may support more consistent reporting and treatment planning in future studies. Further multicenter studies and case aggregations will be essential to validate this approach and possibly define distinct subtypes within this hybrid category.

In this narrative synthesis of the literature on Monteggia injuries, we aimed to contextualize the present case within the broader and heterogeneous spectrum of complex forearm fractures/dislocations. Rather than offering a comprehensive or systematic review, we selectively discuss key studies that highlight the main surgical challenges and evolving treatment principles in this domain.

This case not only illustrates the complexity of managing rare fracture/dislocation patterns but also emphasizes the necessity of integrating osseous and ligamentous repair to restore functional stability. By analyzing this case alongside the existing literature, we derive broader insights into the importance of individualized surgical strategies that address both bone alignment and soft tissue integrity. These considerations may guide future treatment algorithms and contribute to developing more comprehensive classification systems for hybrid injury patterns.

### Study Limitations

This work is based on a single case report, which inherently limits the generalizability of the findings. While the associated narrative literature review helps to contextualize the injury and treatment approach, it does not provide a comprehensive or systematic evaluation of all available evidence. The review is not exhaustive and may be subject to selection bias, as it relies on previously published studies of variable quality.

Additionally, the study is subject to interpretive bias, as the analysis and conclusions are drawn from a single clinical experience without biomechanical validation of the surgical techniques employed. Follow-up imaging was limited to standard radiographs, and more advanced modalities such as CT or MRI were not utilized, potentially restricting the assessment of soft tissue healing and subtle joint changes.

Furthermore, the conclusions drawn from this case are observational in nature and should be interpreted with caution. Prospective studies and multicenter registries would be valuable to further elucidate optimal strategies for managing rare Monteggia variants such as the one described.

## 4. Conclusions

This report presents an exceptionally rare and previously undocumented case of complex forearm instability successfully managed through a single-stage surgical procedure. The surgical approach, carried out step by step, included radial head prosthesis, reconstruction of the ulnar collateral ligament, and fixation of both radial and distal ulnar fractures. Stability was achieved only after addressing all components of the injury, confirming the necessity of a comprehensive and individualized strategy. The particular combination of injuries observed in this casefeatures reminiscent of Monteggia, Galeazzi, and Essex–Lopresti lesions, suggesting the presence of complex hybrid injury patterns that may not be fully encompassed by existing classification systems. While a formal new classification is beyond the scope of this report, awareness and recognition of such atypical patterns are crucial to guide treatment decisions. This case emphasizes the importance of appropriate diagnosis and tailored treatment planning for these complex injury patterns. In light of this, we propose the conceptual term “Hybrid Monteggia Variant” to describe cases that include a proximal ulna fracture with radial head dislocation, together with additional features such as distal radioulnar joint disruption, interosseous membrane injury, or distal forearm fractures. Although not a formal classification, this term may provide a useful framework to improve diagnostic precision, promote shared terminology, and guide multidisciplinary treatment planning.

## Figures and Tables

**Table 1:** Surgical Management and Long-Term Outcomes.

Timing	Findings/Procedures/Outcomes
Pre op	Gross deformity of the left arm, tenderness to the olecranon and wrist regions, and complete functional impairment. Comminuted fracture of the distal third of the radius with dorsal displacement of the distal fragment and a comminuted fracture of the distal ulna metaphysis, and multi-fragmentary radial head fracture with partial detachment of the articular head.
Intraoperative	ORIF of the radial fracture with anatomical locking plate, closed reduction and intramedullary fixation of the ulnar fracture with a Steinmann pin, and prosthetic radial head replacement. Ulnar collateral elbow ligament reconstruction.
Immediate post op (under anesthesia)	Passive flexion up to 130°, extension deficit of 15°, and forearm pronation/supination of 70°/60°. Intraoperative joint stability was satisfactory in both varus and valgus stress.
Early post op	BAM-type plaster splint for 15 days, then protected active and passive mobilization. Targeted physiotherapy protocol was initiated one month after surgery.
2 years post op	Full recovery of elbow flexion and extension. Some limitations in forearm pronation and supination persisted, likely due to her pre-existing condition.

**Table 2:** Literature Summary on Monteggia Variants.

Author	Year	Injury	Treatment	Outcomes
Weber [11]	2022	Monteggia fractures and Monteggia-like lesions	Various techniques for ulnar and radial fractures	Using locking compression plates for ulna fixation could lead to less revision surgery and prevent ulna non-union. Coronoid fractures and reconstructable radial head fractures should be addressed with ORIF.
Tille [12]	2022	Monteggia fractures and Monteggia-like lesions	Ulna: ORIF with locking compression plates. Radial head: osteosynthesis, partial resection, or endoprosthesis. Coronoid process: lag screw or a neutralizing plate	Fractures involving the coronoid process were associated with poorer functional results, while the involvement of the radial head was less prognostically significant.
Klug [13]	2019	Monteggia-like lesions	ORIF, radial head arthroplasty or excision	Choice between osteosynthesis, prosthetic replacement, or resection significantly affects clinical outcomes, especially in comminuted Mason type III fractures.
Jungbluth [14]	2018	Monteggia-like lesions	Ulnar fracture: ORIF with locking compression plate. Radial head fracture: mini screws or cemented bipolar radial head prosthesis. Coronoid fracture: fixation by lag screws inserted through the ulnar plate or by independent lag screws	Careful detection of all elements of the injury, and with appropriate reconstruction, good results can be obtained. Residual postoperative instability: hinged elbow fixator for six weeks to allow healing of the soft tissues. Early postoperative passive motion is recommended to avoid stiffness.
O’Driscoll [15]	2003	Monteggia-like lesions	Stabilize the ulna first, assess radial head alignment, and then address ligamentous injuries, with intraoperative fluoroscopy	N/A

N/A: not applicable.

## References

[1] Bado J.L. (1967). The monteggia lesion. Clin. Orthop. Relat. Res..

[2] Johnson N.P., Silberman M. (2024). Monteggia Fractures. StatPearls [Internet].

[3] Johnson N.P., Smolensky A. (2024). Galeazzi Fractures. StatPearls [Internet].

[4] Hutchinson S., Faber K.J., Gan B.S. (2006). The Essex-Lopresti injury: More than just a pain in the wrist. Can. J. Plast. Surg..

[5] Letts M., Locht R., Wiens J. (1985). Monteggia fracture-dislocations in children. J. Bone Joint Surg. Br..

[6] Ring D., Jupiter J.B. (2002). Fracture-dislocation of the elbow. Hand Clin..

[7] Sajjad Athar M., Ashwood N., Rao V., Galanapoulos I. (2016). Montaggia Type 4 Fracture—A Case Report of an Unusual Presentation of a Rare Injury. Ann. Clin. Case Rep..

[8] Sriramka B., Jain M. (2019). Unusual Forearm Injury. Oman Med. J..

[9] Giannicola G., Sacchetti F.M., Greco A., Cinotti G., Postacchini F. (2010). Management of complex elbow instability. Musculoskelet. Surg..

[10] Masci G., Cazzato G., Milano G., Ciolli G., Malerba G., Perisano C., Greco T., Osvaldo P., Maccauro G., Liuzza F. (2020). The stiff elbow: Current concepts. Orthop. Rev..

[11] Weber M.M., Rosteius T., Schildhauer T.A., Königshausen M., Rausch V. (2023). Monteggia fractures and Monteggia-like-lesions: A systematic review. Arch. Orthop. Trauma. Surg..

[12] Tille E., Seidel L., Schlüßler A., Beyer F., Kasten P., Bota O., Biewener A., Nowotny J. (2022). Monteggia fractures: Analysis of patient-reported outcome measurements in correlation with ulnar fracture localization. J. Orthop. Surg. Res..

[13] Klug A., Konrad F., Gramlich Y., Hofmann R., Schmidt-Horlohé K. (2019). Surgical treatment of the radial head is critical to the outcome of Monteggia-like lesions. Bone Jt. J..

[14] Jungbluth P. (2018). The challenge of Monteggia-like lesions of the elbow: Mid-term results of 46 cases. Bone Jt. J..

[15] O’Driscoll S.W., Jupiter J.B., Cohen M.S., Ring D., McKee M.D. (2003). Difcult elbow fractures: Pearls and pitfalls. Instr. Course Lect..

[16] Ring D., Jupiter J.B., Waters P.M. (1998). Monteggia fractures in children and adults. J. Am. Acad. Orthop. Surg..

[17] Korner J. (2004). Monteggia injuries in adults: Critical analysis of injury pattern, management, and results. Unfallchirurg.

[18] Strauss E.J., Tejwani N.C., Preston C.F., Egol K.A. (2006). The posterior Monteggia lesion with associated ulnohumeral instability. J. Bone Jt. Surg. Br..

[19] Biewener A., Bischoff F., Rischke T., Tille E., Nimtschke U., Kasten P., Schaser K.D., Nowotny J. (2019). Instability of the proximal radioulnar joint in Monteggia fractures-an experimental study. J. Orthop. Surg. Res..

[20] Jupiter J.B., Leibovic S.J., Ribbans W., Wilk R.M. (1991). The posterior Monteggia lesion. J. Orthop. Trauma.

[21] Bowers K.M., Anderson D.E. (2024). Delayed Union and Nonunion: Current Concepts, Prevention, and Correction: A Review. Bioengineering.

